# The Impact of Item Calibration Error on Variable-Length Cognitive Diagnostic Computerized Adaptive Testing

**DOI:** 10.3389/fpsyg.2020.575141

**Published:** 2020-12-02

**Authors:** Xiaojian Sun, Yanlou Liu, Tao Xin, Naiqing Song

**Affiliations:** ^1^School of Mathematics and Statistics, Southwest University, Chongqing, China; ^2^Southwest University Branch, Collaborative Innovation Center of Assessment for Basic Education Quality, Chongqing, China; ^3^China Academy of Big Data for Education, Qufu Normal University, Qufu, China; ^4^Collaborative Innovation Center of Assessment for Basic Education Quality, Beijing Normal University, Beijing, China

**Keywords:** variable-length cognitive diagnostic computerized adaptive testing, calibration errors, classification accuracy, test efficiency, cognitive diagnosis assessment

## Abstract

Calibration errors are inevitable and should not be ignored during the estimation of item parameters. Items with calibration error can affect the measurement results of tests. One of the purposes of the current study is to investigate the impacts of the calibration errors during the estimation of item parameters on the measurement accuracy, average test length, and test efficiency for variable-length cognitive diagnostic computerized adaptive testing. The other purpose is to examine the methods for reducing the adverse effects of calibration errors. Simulation results show that (1) calibration error has negative effect on the measurement accuracy for the deterministic input, noisy “and” gate (DINA) model, and the reduced reparameterized unified model; (2) the average test lengths is shorter, and the test efficiency is overestimated for items with calibration errors; (3) the compensatory reparameterized unified model (CRUM) is less affected by the calibration errors, and the classification accuracy, average test length, and test efficiency are slightly stable in the CRUM framework; (4) methods such as improving the quality of items, using large calibration sample to calibrate the parameters of items, as well as using cross-validation method can reduce the adverse effects of calibration errors on CD-CAT.

## Introduction

Cognitive diagnostic assessment (CDA) has attracted lots of attention because of its advantage that can provide the strengths and weaknesses of examinees for specific content domains rather than just provide an overall score to indicate the position of one examinee relative to others (Leighton and Gierl, 2007). One of the research areas of CDA is cognitive diagnostic computerized adaptive testing (CD-CAT; Cheng, 2009). Compared with paper-and-pencil (P&P) test, CD-CAT can generate suitable tests that match examinees’ latent attribute profiles to produce similar even higher measurement accuracy (Chen et al., 2012; Mao and Xin, 2013; Chang, 2015). Meanwhile, the generated tests have lesser items than the P&P test (Weiss, 1982).

The CD-CAT system has some important components such as item bank, item selection method, psychometric model, and terminal rule, among which item selection method is the key element (Chang, 2015), whereas the item bank is the fundamental factor. A large number of items should be included in the item bank to make sure that all possible latent attribute profiles can be covered. Before the implementation of CD-CAT, the parameters of items should be calibrated; a commonly adopted method is using a limited number of examinees to calibrate these items (Doebler, 2012; van der Linden and Glas, 2000). For now, most studies treated the calibrated parameters of items as their true values, and items were chosen based on their calibrated parameters (Patton et al., 2013). However, studies showed that using a small number of examinees to calibrate items would produce large calibration errors (Şahin and Weiss, 2015). In other words, the estimated parameters of items in the item bank are different from their optimal parameters because of the existence of calibration errors. Researchers have demonstrated that the estimation of examinees’ latent traits would be biased systematically if the calibration errors of items are ignored both in item response theory (IRT)–based CAT and CD-CAT (e.g., Doebler, 2012; Patton et al., 2013; Cheng et al., 2015; Huang, 2018).

Patton et al. (2013) examined the impacts of capitalization on chance on classification accuracy, recovery of ability, and test length in IRT-based variable-length CAT. Results showed that test information would be spuriously overestimated when the calibration sample size is small. In addition, the average test length (ATL) for small calibration sample size was shorter than for large calibration sample size, which indicated that small calibration sample size caused the tests to terminate prematurely (Patton et al., 2013). Huang (2018) investigated the influences of calibration errors on the attribute classification accuracy and measurement precision of attribute mastery classification by using three simulation studies in the context of fixed-length CD-CAT. The author found that calibration errors had negative effect on the classification accuracy and test information when the Deterministic Input Noisy Output “and” Gate (DINA; Junker and Sijtsma, 2001) model was used, while the effect was small for the compensatory reparameterized unified model (CRUM; Templin, unpublished) because of its additive characteristics. In addition, the author also found that using high-quality items, larger sample size, or increasing test length could mitigate the risks of calibration error and increase the measurement accuracy (Huang, 2018).

Although studies conducted by Patton et al. (2013) and Huang (2018) have examined the effects of standard error (SE) on the measurement accuracy and test efficiency for IRT-based variable-length CAT and fixed-length CD-CAT, respectively, the effect of SE on variable-length CD-CAT is still unclear. Compared with fixed-length CD-CAT, the posterior probability of attribute profile was commonly used as the termination rule in variable-length CD-CAT, which became more complicated because the estimation of posterior probability mainly depended on the parameters of items, and the measurement accuracy of attribute profile was strongly related to the posterior probability (Hsu et al., 2013; Hsu and Wang, 2015). The measurement accuracy would be misleadingly estimated due to inaccurate estimates of the posterior probability. For instance, the tests would be terminated prematurely if the spuriously high posterior probability was obtained, as in IRT-based CAT (Huang, 2018). Meanwhile, differed from IRT-based variable-length CAT, which used the conditional SE of ability as the termination rule, the variable-length CD-CAT could not use the SE of attribute profile as the termination rule directly because of the non-computability of the SE. Therefore, the purpose of this study is to investigate the impact of the SE of parameter calibration on the measurement of variable-length CD-CAT.

The rest of the article is organized as follows. First, three kinds of CDMs, which are the DINA model, reduced reparameterized unified model (RRUM; Hartz, 2002), and CRUM, are introduced briefly. Second, an item selection method used in the current study is described. Third, a simulation study is conducted to examine the performance of variable-length CD-CAT under different levels of calibration error. Lastly, discussions and conclusions are provided.

## Introduction of CDMS

### The DINA Model

The DINA model is commonly adopted in CD-CAT framework because of its simplicity (Cheng, 2009; Chen et al., 2012; Mao and Xin, 2013). The model contains two item parameters, which are the guessing and slipping parameters, and can be written as

$$P\left(Y_{i\,j}=1|\eta_{i\,j}\right)={\left(1-s_{j}\right)}^{\eta_{i\,j}}g_{j}^{1-\eta_{i\,j}},$$

where *$\eta_{i\,j}=\prod_{k=1}^{K}{\left(\alpha_{i\,k}\right)}^{q_{j\,k}}$* is the ideal response, which is equal to 1 if the *i*th examinee masters all attributes that item *j* required, and is equal to 0 if at least one of the required attributes of item *j* is missing; *K* is the number of attributes; *α_*ik*_* is the mastery or deficiency of the *k*th attribute for the *i*th examinee; *q*_*jk*_ is the element of the Q-matrix; *g*_*j*_ and *s*_*j*_ are the guessing and slipping parameters for item *j*, respectively.

The DINA model tends to classify examinees into two classes for each item. Specifically, some are those who master all attributes, and others are those who lack of at least one attribute that the item requires, respectively.

### The RRUM

Compared with the DINA model, the RRUM can classify examinees into more than two classes for each item by using different probabilities for different attribute profiles. The RRUM has also attracted considerable attention in CD-CAT in recent years (e.g., Dai et al., 2016; Huebner et al., 2018; Wang et al., 2020) and can be expressed as

$$P\left(Y_{i\,j}=1|\alpha_{i}\right)=\pi_{j}*\prod_{k=1}^{K}r_{j\,k}^{*\left(1-\alpha_{i\,k}\right)\,q_{j\,k}},$$

where $\pi_{j}^{*}$, the baseline parameter, refers to the probability of correct response to item *j* when individuals have mastered all attributes that the item requires; $r_{j\,k}^{*}$, the penalty parameter, denotes the reduction in the probability of correct response to item *j* when an individual lacks attribute *k*. Both $\pi_{j}^{*}$ and $r_{j\,k}^{*}$ range from 0 to 1.

### The CRUM

Both of the DINA model and the RRUM are non-compensatory CDMs; the CRUM, on the contrary, is the compensatory model. The probability of correctly answering an item is defined as the addition of intercept and main effect of attributes that the item requires by using the logit as the link function (Templin, unpublished). The item response function of the CRUM can be written as

$$P\left(Y_{i\,j}=1|\alpha_{i}^{*}\right)=\frac{\exp\left(\delta_{j\,0}+\sum_{k=1}^{K_{j}^{*}}\delta_{j\,k}\,\alpha_{i\,k}\,q_{j\,k}\right)}{1+\exp\left(\delta_{j\,0}+\sum_{k=1}^{K_{j}^{*}}\delta_{j\,k}\,\alpha_{i\,k}\,q_{j\,k}\right)},$$

where δ_*j0*_ is the intercept, which refers to the probability of correctly answering item *j* when none of the required attributes is mastered; δ_*jk*_ is the main effect of attribute *k*, representing the change in probability when attribute *k* is mastered, and $K_{j}^{*}$ is the number of attributes that item *j* requires.

## Item Selection Method

A number of item selection methods have been developed in CD-CAT, for instance, the Kullback–Leibler (KL) and the Shannon entropy (SHE; Xu et al., 2003) strategies, the posterior-weighted KL (PWKL; Cheng, 2009) strategy, the modified PWKL (MPWKL) and the generalized DINA (GDINA; de la Torre, 2011) model discrimination index (GDI) strategies (Kaplan et al., 2015), and the mutual information (MI; Wang, 2013) strategy. Among these strategies, both of MI and MPWKL strategies can produce high measurement accuracy when the test length is short; in addition, the MPWKL strategy performs better than the traditional WPKL method (Kaplan et al., 2015). The MPWKL strategy is commonly used in CD-CAT (e.g., Huang, 2018; Huebner et al., 2018; Wang et al., 2020); therefore, it will be adopted in current study. The expression of the MPWKL for item *j* can be formulated as

$$MPWKL_{i\,j}=\sum_{d=1}^{2^{K}}\{\sum_{c=1}^{2^{K}}[\sum_{x=0}^{1}\log\left(\frac{P\left(Y_{i\,j}=x|\alpha_{d}\right)}{P\left(Y_{i\,j}=x|\alpha_{c}\right)}\right)$$

$$\;P\left(Y_{i\,j}=x|\alpha_{d}\right)\pi \left(\alpha_{c}|Y_{i,n-1}\right)]\pi \left(\alpha_{d}|Y_{i,n-1}\right)\},$$

where *P*(*Y*_*ij*_ = *x*|α) is the item response function, *Y*_*i,n–1*_ is the response vector for the first *n*—1 items for the *i*th examinee, and π(α|*Y*_*i*,*n*−1_) is the posterior probability for attribute profile ***α***; the rests are defined as above. Items with the largest MWPKL index will be selected during the implementation of CD-CAT.

## Simulation Study

### Independent Variables

Several factors are manipulated in this study, including model type, quality of the item bank, magnitude of calibration error, and termination rule. Specifically, three CDMs, which are the DINA model, RRUM, and CRUM, are adopted in this study, and these models have been used in previous studies (e.g., Chen et al., 2012; Mao and Xin, 2013; Wang, 2013; Huang, 2018; Huebner et al., 2018; Wang et al., 2020). In addition, three levels are manipulated for the quality of the item bank, which are high-, low-, and mix-item quality (i.e., the mix of high- and low-quality items), respectively, and the corresponding item parameters are listed in Table 1. All these settings are modified from previous studies (Chen et al., 2012; Huang, 2018; Wang et al., 2020). As for the magnitude of calibration error, similar with Huang (2018), four levels are adopted in this study, while the specific values are different. A pilot study shows that when the models are the DINA model and the RRUM in CD-CAT, the standard deviations (SDs) of the calibration errors are smaller than 0.1 for different calibration sample sizes conditional on high-quality items. Considering that the SD might be larger than 0.1 when the quality of items is low, the SD values of calibration errors are 0, 0.1, 0.2, and 0.3, which are named true value, small, median, and large errors, respectively. The mean value is set as 0 for all the calibration errors. Consistent with previous studies (Hsu et al., 2013; Hsu and Wang, 2015), the posterior probability of attribute profile is used as the termination rule, and the termination criteria are 0.7 and 0.8, respectively.

**TABLE 1:** Simulation design of the current study.

**Independent variable**		**Values**
Model		DINA, RRUM, CRUM
Calibration error		0, 0.1, 0.2, 0.3
Termination rule		0.7, 0.8
Item quality	High	DINA: *s*, *g* ∼ *U* (0.05, 0.25)
		RRUM: *π^∗^* ∼ *U* (0.75, 0.95); *r*^∗^ ∼ *U* (0.05, 0.40)
		CRUM: *δ_*0*_* ∼ *U* (−3, −1.1); *P*(*Y*_*j*_ = 1)∼ *U* (0.85, 1.0)
	Low	DINA: *s*, *g* ∼ *U* (0.25, 0.45)
		RRUM: *π^∗^* ∼ *U* (0.55, 0.75); *r*^∗^ ∼ *U* (0.15, 0.50)
		CRUM: *δ_*0*_* ∼ *U* (−1.1, −0.2); *P*(*Y*_*j*_ = 1)∼ *U* (0.6, 0.85)
	Mix	DINA: *s*, *g* ∼ *U* (0.05, 0.45)
		RRUM: *π^∗^* ∼ *U* (0.55, 0.95); *r*^∗^ ∼ *U* (0.05, 0.50)
		CRUM: *δ_*0*_* ∼ *U* (−3, −0.2); *P*(*Y*_*j*_ = 1)∼ *U* (0.6, 1.0)

*P(Y_j_ = 1) refers to the probability of correctly answering item j.*

### Control Variables

Five variables are fixed in current study. Specifically, 5 attributes and 300 items, which are commonly adopted in empirical and simulation studies (e.g., Liu et al., 2013; Mao and Xin, 2013; Huang, 2018; Huebner et al., 2018; Wang et al., 2020), are used in this study. The generation of Q-matrix is consistent with Chen et al. (2012). Specifically, three basic Q-matrices, which include items measuring one, two, and three attribute(s), respectively, are generated, namely Q_1_, Q_2_, and Q_3_, respectively. Then, the final Q-matrix is constructed by merging 20 basic matrix Q_1_, 14 basic matrix Q_2_, and 6 basic matrix Q_3_. In addition, the number of examinees is set as 2,000, which is consistent with Mao and Xin (2013) and Huang (2018). The generation of the attribute profile is consistent with Cheng (2009), which assumes that the probability that each examinee masters each attribute is 0.5. Meanwhile, the item selection method is MPWKL, and the maximum number of items of the test is 30, which are adopted in previous studies (Huebner et al., 2018).

There are 3 × 3 × 4 × 2 = 72 conditions in total, among which the termination rule is within-group variable, and the rest are between-group variables. Twenty-five replications are generated for each condition to reduce the sample error. The simulation study is implemented in R software, and the codes are available upon request from the corresponding author.

### Measurement Criteria

Two indices are used to evaluate the measurement accuracy of attribute classification, which include pattern correct classification rate (PCR) and average attribute correct classification rate (AACR). These two indices both range from 0 to 1, and larger value indicates better performance. They can be expressed as

$$PCR=\sum_{i=1}^{N}I\left({\hat{\alpha }}_{i}=\alpha_{i}\right)/N,$$

$$AACR=\sum_{i=1}^{N}\sum_{k=1}^{K}I\left({\hat{\alpha }}_{i\,k}=\alpha_{i\,k}\right)/\left(N\times K\right),$$

where *N* is the number of examinees; *I*(⋅) is the indicator function, which equals 1 if ${\hat{\alpha }}_{i}=\alpha_{i}$(or${\hat{\alpha }}_{i\,k}=\alpha_{i\,k}$) is true, and vice versa; ${\hat{\alpha }}_{i}$ is the estimate of attribute profile, and α_*i*_ is the true attribute profile.

In addition to the evaluation of the measurement accuracy, the relative measurement precision (RMP) index is also used to evaluate the measurement precision of the estimated attribute profile (Huang, 2018). Moreover, based on the study conducted by Patton et al. (2013), the relative test efficiency (RTE) is used to evaluate the test efficiency in CD-CAT. These two indices can be written as

$$R\,M\,P=\frac{\sum_{i=1}^{N}\max_{c=1,2,\cdots ,2^{K}}\left(\pi \,\left(\alpha_{c}|Y_{i},\hat{\delta }\right)\right)}{\sum_{i=1}^{N}\max_{c=1,2,\cdots ,2^{K}}\left(\pi \,\left(\alpha_{c}|Y_{i},\delta \right)\right)}\,\text{ and}$$

$$R\,T\,E=\frac{\sum_{i=1}^{N}\sum_{j=1}^{J_{i}}\hat{C\,D\,I_{j}}}{\sum_{i=1}^{N}\sum_{j=1}^{J_{i}}C\,D\,I_{j}},$$

where $\hat{\delta }$ and δ are vectors including all estimated and true item parameters that examinee *i* answers, respectively. CDI is the cognitive diagnostic index and proposed by Henson and Douglas (2005), which can be regarded as the test information for each examinee in current study. The details can be found in Henson and Douglas (2005).

## Results

**FIGURE 1:**
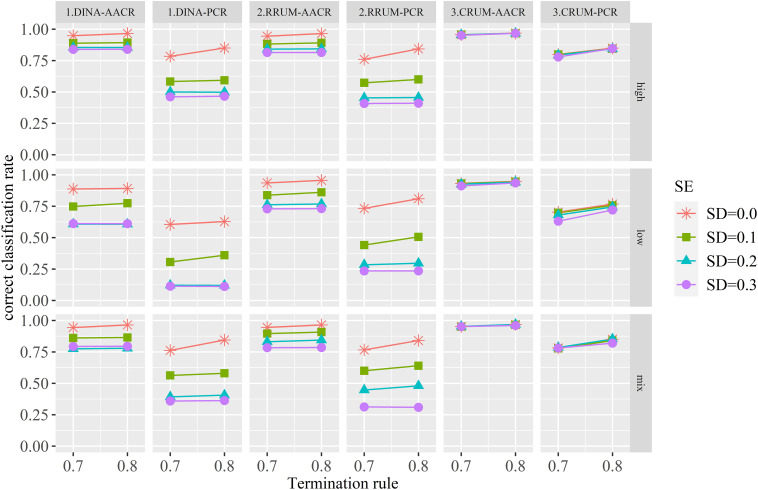
The correct classification rates for all conditions.

Figure 1 presents the estimated AACR and PCR for each condition. Some meaningful findings can be summarized from the figure. First, the calibration error has negative effect on the AACR and PCR for the DINA model and RRUM irrespective of the item quality and termination rule. With the increase in SD of calibration error, the AACR and PCR decrease for these two models. The effect of calibration error on CRUM can be ignorable, especially for high- and mix-item quality. The differences between the true value (*SD* = 0) and calibration errors are small enough. Second, for the DINA model, the differences of AACR and PCR between median (*SD* = 0.2) and large (*SD* = 0.3) are small, regardless of the item quality and termination rule. The same results are found for the RRUM under high- and low-item quality, whereas the differences are large when the item bank is of mixed quality. Third, the quality of the item bank has positive effect on PCR, regardless of model type and termination rule. Items with high quality produce higher PCRs than low-quality items. Fourth, termination rule has a positive effect on PCR for the CRUM, whereas the effects are complicated for the DINA model and RRUM. When calibration error is zero, termination rule has positive effect on PCR, while the positive effect of termination rule on PCR tends to decrease with the increase of calibration error. Fifth, the PCRs and AACRs are stable for all conditions, and the SDs of PCR for the DINA model, RRUM, and CRUM range from 0.005 to 0.013, 0.008 to 0.015, and 0.007 to 0.013, respectively. Meanwhile, the SDs of AACR range from 0.006 to 0.049, 0.006 to 0.044, and 0.004 to 0.016, respectively, for these three CDMs.

**FIGURE 2:**
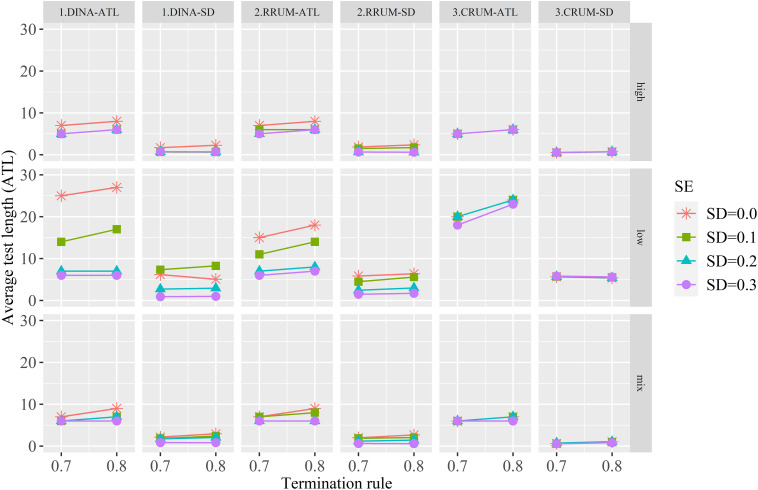
The average test length and the corresponding SDs for all conditions.

Figure 2 shows the ATL and the corresponding SD. Some meaningful points are summarized. First, the true value (*SD* = 0) produces the longest ATL for the DINA model and RRUM, and with the increase of calibration error, the ATL, in general, tends to decrease, especially for the low-quality item. The CRUM, on the contrary, produces similar ATL irrespective of calibration error. Second, the quality of the item bank has an effect on ATL. Items with high and mixed quality tend to produce shorter ATL than low-quality items, and high- and mixed-quality items produce similar ATL. Third, termination rule has positive effect on ATL, especially for true and small calibration error. The ATLs are relatively stable for median and large calibration error. These results indicate that items with calibration error tend to terminate prematurely for the DINA model and RRUM. The CRUM, on the contrary, is not affected by calibration error.

**FIGURE 3:**
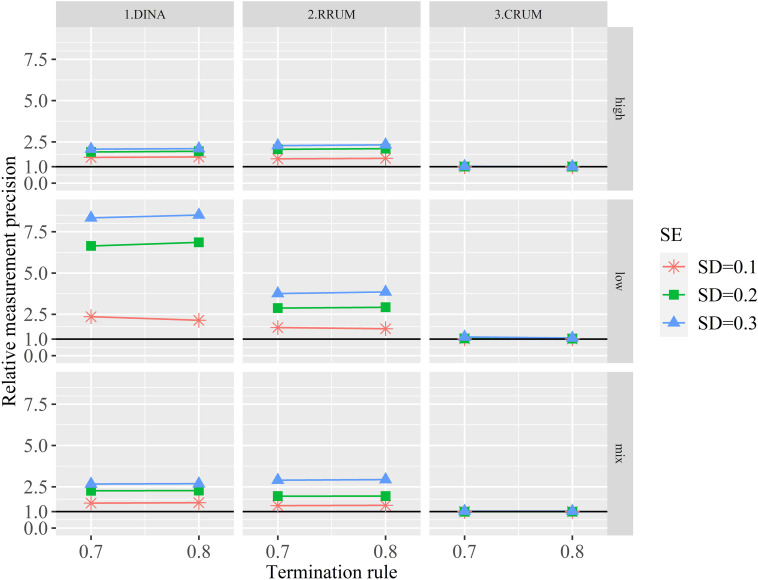
The relative test efficiency with different calibration errors.

Figure 3 depicts the RMPs with different calibration errors. The RMPs are greater than one in all conditions. Specifically, small calibration error produces the smallest RMPs for the DINA model and RRUM irrespective of the item quality and termination rule. The large calibration error, on the contrary, produces the largest RMPs. In other words, items with calibration error can produce spuriously high measurement precision, indicating that the posterior probability that an examinee belongs to a specific attribute profile is overestimated substantially, especially for low-quality items. By contrast, the RMPs are closed to one for the CRUM, regardless of the item quality and termination rule, suggesting that the CRUM can produce similar measurement precision between true and calibration error–affected items.

Table 2 lists the RTE for each calibration error. The results are similar to those for the RMP. Overall, the smaller the calibration error is, the better the RTE is for the DINA model and RRUM, regardless of the item quality and termination rule. On the contrary, the calibration error has ignorable effect on the RTE for the CRUM. In addition, the calibration error has more serious effect on the DINA model than the RRUM; the RTE indices are larger than those for the RRUM.

**TABLE 2:** The relative test efficiency for each calibration error.

**Model**	**Item quality**	**Calibration error**	***ε* = 0.7**		***ε* = 0.8**	
			***M***	***SD***	***M***	***SD***
DINA	Low	0.1	2.456	0.016	2.299	0.029
		0.2	18.782	0.200	19.528	0.259
		0.3	45.504	0.703	45.149	0.460
	High	0.1	5.149	0.034	5.117	0.039
		0.2	6.752	0.050	6.777	0.047
		0.3	8.442	0.064	8.408	0.049
	Mix	0.1	4.008	0.026	3.998	0.035
		0.2	5.160	0.055	5.346	0.053
		0.3	11.294	0.069	11.251	0.076
RRUM	Low	0.1	1.778	0.015	1.683	0.013
		0.2	6.112	0.061	6.008	0.072
		0.3	9.882	0.100	9.617	0.093
	High	0.1	3.386	0.018	3.444	0.019
		0.2	5.968	0.040	5.937	0.035
		0.3	6.120	0.028	6.134	0.039
	Mix	0.1	3.112	0.025	2.885	0.025
		0.2	5.005	0.036	5.106	0.034
		0.3	6.802	0.060	6.766	0.082
CRUM	Low	0.1	1.019	0.002	1.017	0.002
		0.2	1.041	0.004	1.026	0.004
		0.3	1.076	0.006	1.054	0.008
	High	0.1	1.007	0.000	1.005	0.000
		0.2	1.013	0.001	1.006	0.001
		0.3	1.065	0.001	0.983	0.001
	Mix	0.1	1.007	0.000	1.013	0.000
		0.2	0.997	0.001	0.978	0.001
		0.3	1.098	0.002	1.059	0.001

## Discussion

CD-CAT can diagnose the strengths and weaknesses of examinees with fewer items and provide feedback immediately (Chen et al., 2012; Mao and Xin, 2013; Chang, 2015; Wang et al., 2020). Before the implementation of CD-CAT, parameters of items in the bank need to be calibrated. However, a limited number of examinees are commonly used to calibrate the item parameters to maintain the security of the item bank; therefore, calibration errors exist in these items (van der Linden and Glas, 2000; Doebler, 2012; Patton et al., 2013; Huang, 2018). The current study investigates the impacts of calibration error on variable-length CD-CAT, as well as examines the methods for reducing the adverse effects of calibration errors. Simulation study indicates that calibration error has substantial effect on the classification accuracy, the ATL, measurement precision, and test efficiency. Meanwhile, several factors are found that can be used to reduce the adverse effects of calibration errors.

Results show that for the DINA mode and the RRUM, with the increase of calibration error, the correct classification rates are decreasing, suggesting that the calibration error should not be ignored when CD-CAT is used to diagnose the mastery of examinees, or the results will be inaccuracy, which is consistent with previous studies (Patton et al., 2013; Huang, 2018). At the same time, compared with true item parameter, the ATL becomes shorter for these error-affected item parameters. In other words, when true item parameters are used to select candidate items, to obtain the prespecified posterior probability of attribute profile, more items are needed. However, fewer items are sufficient to obtain the prespecified posterior probability for these error-affected item parameters. Such results indicate that the error-affected item parameters would terminate the test prematurely for examinees, and the similar results were found in IRT-based CAT conducted by Patton et al. (2013).

In addition, the results based on the RMP demonstrate that calibration error tends to produce spuriously high posterior probability that an examinee belongs to a specific attribute profile, which can be used to explain why the test is terminated prematurely for error-affected item parameters. In variable-length CD-CAT, the posterior probability is mainly used as the termination rule, which is adopted in current study as well. When items with calibration error are used in the test, the overestimated posterior probability is obtained; therefore, it is easy to meet the prespecified termination criteria; consequently, the test would be ended. Huang (2018) also obtained the similar result in the context of fix-length CD-CAT. Moreover, the results of the RTE indicate that the calibration error-affected items produce overestimated test information. This finding is consistent with Patton et al. (2013), but inconsistent with Huang (2018). Huang (2018) found that larger calibration errors produce lower CDI values. One possible explanation for this inconsistence is that the ways of calculating the CDI are different. In current study, the CDI values are calculated based on the same items, but the parameters are different, while both of the items and item parameters are different in Huang’s (2018) study.

The results based on the CRUM show that calibration error, in general, has ignorable effects on the attribute classification accuracy, ATL, measurement precision, and test efficiency, regardless of the item quality and termination rule. The classifications of attribute and attribute profile are relatively higher for the CRUM than for the DINA model and the RRUM. The similar results are found in Huang’s (2018) study. In addition, item quality has a negative effect on ATL for the CRUM, which is low-quality item that produces the longest test length. The differences of ATL between high and mixed quality are comparable.

In summary, compared with the DINA and RRUM, the presence of calibration error does not affect the measurement accuracy of the CRUM, and the possible reason can be attributed to the additive characteristic of this model (Huang, 2018). For instance, some parameters may be overestimated, whereas others may be underestimated for a specific item, and at this point, the summon of these parameters may be consistent with the true value because of the counterbalance of the overestimated and underestimated parameters. The DINA model and RRUM, on the contrary, do not possess the additive characteristic. Specifically, the probability of answering correctly involves only one parameter (the slipping or guessing) for the DINA model; thus, the result would be inconsistent with the true value if the calibration error is considered. Meanwhile, the characteristic of the RRUM is multiplicative property rather than additive property; therefore, the multiplication of the baseline and penalty parameters contaminated by calibration error may produce the final result that is different from the true value of the item.

According to the results of the simulation study, some factors can be summarized to reduce the adverse effect of calibration error on variable-length CD-CAT. First, considering that there is negative relationship between calibration sample size and calibration error of items (Huang, 2018), therefore increasing the sample size of calibration should be a feasible way to achieve this purpose. Second, compared with the low- and mixed-quality items, the differences between contaminated by calibration error and true item parameters are relatively smaller conditional on the high-quality items, which means improving the quality of the item bank is another way to achieve this purpose. In addition to these factors, previous studies indicated that increasing the test length could also relieve the negative effect of the calibration error (Patton et al., 2013; Huang, 2018). Moreover, the cross-validation method, which is used by Patton et al. (2013) in the CAT framework, can also reduce the adverse effect of calibration error. Results based on four conditions, including the cross combination of the quality of the item bank (low and high quality) and the SD of calibration error (0.2 and 0.3), show that using the cross-validation method can produce better performance of the measurement accuracy, RMP, and RTE than the method without using cross-validation. Furthermore, Huang (2018) also found that using the item exposure control method, such as the Sympson and Hetter on line method with freeze (SHOF method), could also relieve the negative impact of calibration error. The item selection strategy with the SHOF method produces slightly lower classification accuracy than strategy without SHOF method, while the values of the RMP produced by strategy with the SHOF method are close to one for low-quality items (Huang, 2018).

Although some promising results are obtained in this study, some possible directions can be investigated for future studies. For instance, the complexity of Q-matrix has an important effect on classification accuracy and is not considered in this study. Future study can be conducted to explore the performance of this factor. In addition, the setting of calibration error may not represent the empirical situation sufficiently; therefore, using different calibration sample size to calibrate the item parameters may be an option for future study. Moreover, only MPWKL strategy is used in the current study; other item selection strategies such as MI and SHE strategies should be adopted in the future. Furthermore, all CDMs used in the current study are special cases of the GDINA model and the log-linear cognitive diagnostic model (LCDM; Henson et al., 2009). Compared with the GDINA model and LCDM, the CDMs used in this study lack flexibility and have more restrictions (Rupp et al., 2010). Therefore, general CDMs such as the GDINA model and LCDM should be investigated in the future.

## Data Availability Statement

The raw data supporting the conclusions of this article will be made available by the authors, without undue reservation, to any qualified researcher.

## Author Contributions

XS, TX, and NS proposed the original concept and designed the fundamental study of this study. XS and YL wrote the simulation study code and organized the article. All authors contributed to the manuscript revision.

## Conflict of Interest

The authors declare that the research was conducted in the absence of any commercial or financial relationships that could be construed as a potential conflict of interest.
